# The Pan-American Medical Congress

**Published:** 1893-03

**Authors:** M. H. Fletcher

**Affiliations:** Executive President of the Section on Oral and Dental Surgery of the P. A. M. C.


### Domestic Correspondence.

### THE PAN-AMERICAN MEDICAL CONGRESS.

### SECTION ON ORAL AND DENTAL SURGERY.

CINCINNATI, OHIO, January 31, 1893.

TO THE DENTAL PROFESSION OF THE WESTERN CONTINENT:

The Pan-American Medical Congress, to meet in Washington, D. C., September 5, 6, 7, and 8, 1893, being an assured success, the Dental Section promises to be well represented.

No other section of the Congress can claim a greater number of men of scientific attainment. In artistic and mechanical skill, in accurate and delicate manipulation where surgery is involved, in bacteriology and histology and rapid progress in its specialty, no other surpasses that of the dental profession. Able papers on the following subjects will attest the above assertion: Cleft Palate, Hare-lip, Orthodontia, Dental Anatomy, Histology and Pathology, New Growths of every character pertaining to the mouth and teeth, Diseases of the Maxillary Sinus and Alveolar Processes, Periostitis, Pulpitis, and their Results, Operative Dentistry, Bacteriology, Mechanical Dentistry, in addition to many other suitable topics.

This Congress being an outgrowth of the American Medical Association, the requirements for membership in this section are identical with that of the A.M.A.,—viz., any reputable practitioner holding the title of D.D.S. or M.D.S. can become a member the same as if he possessed the M.D.

To members of the profession in our sister countries we extend a hearty invitation to visit us and participate in the meeting, either by writing papers or by being present to hear or discuss them. This is especially desirable, since the Congress belongs equally to all American countries. Many of you will, no doubt, visit the great World's Fair. This is also the year for the World's Columbian Dental Congress in Chicago. This meeting, however, in no way interferes with the P. A. M. C., since the Columbian comes August 14 to 19, inclusive, and the P. A. M. C. September 5 to 18, inclusive, in Washington, thus affording two attractions in the way of scientific dentistry in addition to the great Fair. Many of the officers of one Congress are officially connected with the other.

To the Columbian you are an invited guest; at the Pan-American you are participating in an institution as much your own as ours.

To the dental profession in the United States we would suggest that in taking part in this, the first meeting of the P. A. M. C., we are the hosts, and our duties as such need not be rehearsed.

The excess of dental practitioners in the United States over our sister countries will necessitate a careful selection of topics and papers in order to present the highest standard, the object being not numbers, but quality of material and ample time for discussion.

The social feature of the Congress will be no small part of the attractions.

Respectfully,

M. H. Fletcher, R. P.,

*Executive President of the Section on Oral and Dental Surgery of the P. A. M. C.*

